# BRD4 regulates adiponectin gene induction by recruiting the P-TEFb complex to the transcribed region of the gene

**DOI:** 10.1038/s41598-017-12342-2

**Published:** 2017-09-20

**Authors:** Naoko Sakurai, Yuko Inamochi, Takuya Inoue, Natsuyo Hariya, Musashi Kawamura, Masami Yamada, Anup Dey, Akira Nishiyama, Takeo Kubota, Keiko Ozato, Toshinao Goda, Kazuki Mochizuki

**Affiliations:** 1 Laboratory of Nutritional Physiology, Graduate School of Nutritional and Environmental Sciences, University of Shizuoka, Shizuoka, Japan; 2 Division of Engineering, Interdisciplinary Graduate School of Medicine and Engineering, University of Yamanashi, Yamanashi, Japan; 3 Department of Nutrition, Faculty of Health and Nutrition, Yamanashi Gakuin University, Yamanashi, Japan; 4 Laboratory of Food and Nutritional Sciences, Department of Local Produce and Food Sciences, Faculty of Life and Environmental Sciences, University of Yamanashi, Yamanashi, Japan; 5 Laboratory of Molecular Growth Regulation, NICHD, NIH, Bethesda, MD USA; 6 Department of Child Studies, Faculty of Child Studies, Seitoku University, Chiba, Japan

## Abstract

We previously reported that induction of the adipocyte-specific gene adiponectin (*Adipoq*) during 3T3-L1 adipocyte differentiation is closely associated with epigenetic memory histone H3 acetylation on the transcribed region of the gene. We used 3T3-L1 adipocytes and *Brd4* heterozygous mice to investigate whether the induction of *Adipoq* during adipocyte differentiation is regulated by histone acetylation and the binding protein bromodomain containing 4 (BRD4) on the transcribed region. Depletion of BRD4 by shRNA and inhibition by (+)-JQ1, an inhibitor of BET family proteins including BRD4, reduced *Adipoq* expression and lipid droplet accumulation in 3T3-L1 adipocytes. Additionally, the depletion and inhibition of BRD4 reduced the expression of many insulin sensitivity-related genes, including genes related to lipid droplet accumulation in adipocytes. BRD4 depletion reduced P-TEFb recruitment and histone acetylation on the transcribed region of the *Adipoq* gene. The expression levels of *Adipoq* and fatty acid synthesis-related genes and the circulating ADIPOQ protein level were lower in *Brd4* heterozygous mice than in wild-type mice at 21 days after birth. These findings indicate that BRD4 regulates the *Adipoq* gene by recruiting P-TEFb onto acetylated histones in the transcribed region of the gene and regulates adipocyte differentiation by regulating the expression of genes related to insulin sensitivity.

## Introduction

Recent advances have demonstrated that adipose tissues function as endocrine organs by secreting adipocytokines and hormones, as well as being organs for energy storage. Normal adipocytes with sufficient insulin sensitivity have a great capacity for incorporating glucose and fatty acids through the actions of insulin, and secrete various adipocytokines, including adiponectin (ADIPOQ) and leptin^1^. In particular, ADIPOQ has beneficial functions in normalizing glucose and lipid metabolism in many peripheral tissues. Indeed, ADIPOQ reduces plasma glucose and triacylglycerol levels by enhancing glucose incorporation into adipose tissues and skeletal muscles^2^, activating β-oxidation, and repressing fatty acid synthesis in the liver^3^. Conversely, tumour necrosis factor-α, interleukin-6^4^, and resistin^5^ are secreted by adipocytes with insulin resistance, whereby incorporation of glucose and fatty acids into the cells is reduced through the less efficient actions of insulin. The secretion of these proinflammatory cytokines is associated with obesity, hypertriglyceridemia, and type 2 diabetes and their complications. Notably, the circulating ADIPOQ concentration is inversely associated with these diseases. Thus, control of the function of adipocytes is considered important, particularly to maintain a higher circulating concentration of ADIPOQ protein to prevent abnormalities in glucose and lipid metabolism.

To prevent or control the above diseases, many scientists have been intensively studying the molecular mechanisms underlying the changes in gene expression, in particular in ADIPOQ expression, in adipocytes according to their state of insulin sensitivity and/or adipocyte differentiation. Cell culture studies have already demonstrated that the expression of many genes related to insulin sensitivity, such as adipocyte fatty acid-binding protein (*Albp*)^6^, glucose transporter 4 (*Glut4*)^7^ and *Adipoq* ^8^, are induced during adipocyte differentiation. Several recent studies have shown that differentiation and gene expression in adipocytes are regulated by certain transcription factors, such as cAMP response element-binding protein (CREB), CCAAT/enhancer-binding protein (C/EBP) family members, and peroxisome proliferator-activated receptor (PPAR) γ^9^. Notably, a subtype of PPARγ, PPARγ2, has been shown to be important for adipocyte differentiation, as PPARγ2 transfection into mouse fibroblasts induces adipocyte differentiation^10^. These transcription factors activate the expression of their target genes through binding to cis-regulatory elements located on the promoter/enhancer regions of the genes, and then enhance the recruitment of mRNA transcription initiation complexes onto the promoter/enhancer regions of the genes.

Obesity and type 2 diabetes are caused by long-term lifestyle-associated factors and are suggested to be predisposed to by acquired changes that are not written into the genetic code, designated “epigenetic memory”^11^. In particular, histone tail modifications are important for transcription regulation variation by lifestyle factors. Multiple lysine or arginine residues in the core histones, particularly histones H3 and H4, are subjected to posttranslational modifications, including methylation and acetylation, and many of these modifications are associated with distinct transcription states. Hyperacetylation of histones H3 and H4 is not only associated with the emergence/frequency of euchromatin regions on the genome, but also induces transcription by recruiting transcriptional complexes onto the target genes^12^. Many studies have suggested that acetylation of histones leads to target gene induction by controlling the accessibility of the transcriptional initiation machinery, including bromodomain proteins that bind to acetylated histones, and to chromatin with acetylated histones^13^. It has been reported that induction of the *Pparγ2* gene during adipogenesis is associated with both induction of histone acetylation and one of the bromodomain proteins, BRG1, which is a component of the SWI/SNF complex, in the promoter region of the *Pparγ2* gene^14^. In addition, our previous study showed that both acetylation of histone H3 at lysine 9 near the *Adipoq* gene and its expression were enhanced during adipocyte differentiation. Notably, acetylation was strongly induced in the transcribed region as well as in the promoter/enhancer region during differentiation^15^. However, the biological significance of histone acetylation in the transcribed region of the *Adipoq* gene was not assessed.

Recent studies have demonstrated that one of the bromodomain proteins, BRD4, regulates the mRNA transcription elongation step by enhancing RNA polymerase II C-terminal domain phosphorylation at the serine 2 residue by recruiting the CYCLIN T1-cyclin-dependent kinase (CDK) 9 complex, which is also known as positive transcription elongation factor b (P-TEFb), onto the transcribed region of its target genes^16,17^. Microarray analysis showed that BRD4 knockdown by shRNA reduced global gene expression by approximately 10% during the G1–S transition in NIH3T3 cells^17^. Furthermore, *Brd4* heterozygous mice show reduced adipose tissue weight compared with wild-type mice^18^. These findings suggest that BRD4 and P-TEFb may be important for both adipocyte gene expression and differentiation. However, whether the expression of each functional gene in differentiated adipocytes is regulated by a transcription elongation reaction triggered by acetylated histones-BRD4-P-TEFb has not been examined.

In this study, we examined whether BRD4 regulates *Adipoq* gene expression by recruiting P-TEFb onto the transcribed region of the gene, and whether BRD4 regulates the expression of other genes related to insulin sensitivity along with adipocyte differentiation in 3T3-L1 adipocytes. Furthermore, we performed gene targeting in mice to investigate whether BRD4 regulates the expression of *Adipoq* and insulin sensitivity-related genes during postnatal development.

## Results

### Effects of BRD4 depletion and inhibition on *Adipoq* mRNA and protein levels and lipid droplet accumulation in 3T3-L1 adipocytes

**Figure 1:**
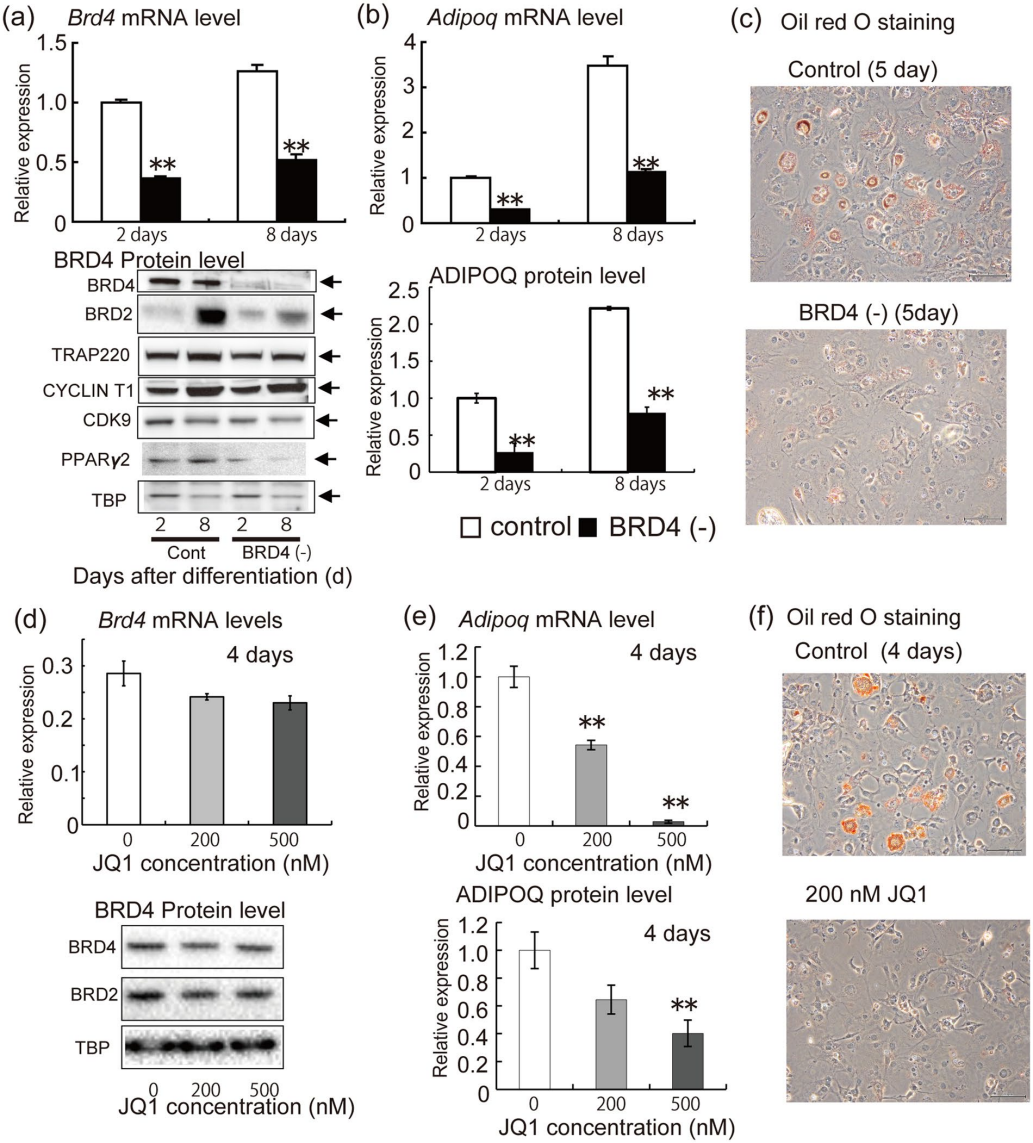
Effects of BRD4 depletion and inhibition on mRNA and protein expression of *Adipoq* and lipid droplet accumulation in 3T3-L1 adipocytes. (**a**) mRNA levels of *Brd4* and protein levels of BRD4, BRD2, TRAP220, CYCLIN T1, CDK9, PPARγ2, and TATA box-binding protein (TBP) at 2 and 8 days after differentiation stimulation. Control or *Brd4* shRNA-expressing 3T3-L1 cells were treated with medium for differentiation. (**b**) Levels of *Adipoq* mRNA in cells and ADIPOQ protein in medium at 2 and 8 days after differentiation stimulation. (**c**) Lipid droplet accumulation at 5 days after differentiation stimulation (Oil red analysis). (**d**) *Adipoq* mRNA in 3T3-L1 adipocytes, secreted ADIPOQ protein in the medium, and protein levels of BRD4, BRD2 and TBP in cells. (+)-JQ1 treatment of 3T3-L1 adipocytes was performed for 4 days from the adipocyte differentiation. (**e**) Lipid droplet accumulation (Oil red O analysis). The data shown are means ± SEM of 6 wells per condition. **P < 0.01 versus the corresponding control cells by Student’s *t*-test (**a**,**b**) or Dunnett’s test based on analysis of variance (**d**).

We constructed BRD4-depleted 3T3-L1 cells using shRNA and a stable retrovirus infected cell line system. *Brd4* mRNA and protein levels at 2 and 8 days after differentiation stimulation were lower in *Brd4* shRNA-expressing cells than in control shRNA-expressing cells. Protein expression levels of TRAP220, CYCLIN T1, CDK9 and TBP were not remarkably different between *Brd4* shRNA-expressing cells and control shRNA-expressing cells at days 2 and 8 after differentiation stimulation. BRD2 expression was higher at 2 days than at 8 days in control cells, while the *Brd4* shRNA treatment reduced the BRD2 protein expression at 8 days (Fig. 1a). A signal for BRD3 was not detected (data not shown). PPARγ2 protein levels were lower in *Brd4* shRNA-expressing cells at 2 and 8 days after differentiation stimulation than in control shRNA-expressing cells. *Adipoq* mRNA in cells and secreted protein in medium were lower in *Brd4* shRNA-expressing cells at 2 and 8 days after differentiation stimulation than in control shRNA-expressing cells (Fig. 1b). Lipid droplet accumulation evaluated by Oil red O staining at 5 days after differentiation stimulation was lower in *Brd4* shRNA-expressing cells than in control shRNA-expressing cells (Fig. 1c).

Next, we examined whether treatment with (+)-JQ1, an inhibitor of the association between acetylated histones and the bromodomain and extra-terminal (BET) family proteins including BRD4, altered *Adipoq* mRNA and protein expression and lipid droplet accumulation in 3T3-L1 adipocytes. mRNA and protein expression levels of BRD4, BRD2 and TBP were not remarkably altered by the (+)-JQ1 treatment (Fig. 1d). Again, signals for BRD3 were not detected (data not shown). In contrast, *Adipoq* mRNA levels in cells were significantly reduced by 200 and 500 nM (+)-JQ1 treatment at 4 days after differentiation. ADIPOQ protein level in medium was reduced by 500 nM (+)-JQ1 treatment at 4 days after differentiation (Fig. 1e). Furthermore, treatment with 200 nM (+)-JQ1 after differentiation stimulation for 5 days reduced lipid droplet accumulation as evaluated by Oil red O staining (Fig. 1f).

Taken together, these results show that BRD4 depletion or inhibition reduced *Adipoq* mRNA levels in cells and protein levels in medium, as well as lipid droplet accumulation, in 3T3-L1 adipocytes.

### Effects of BRD4 depletion on acetylated histones and P-TEFb, PPARγ2 and TRAP220 binding around the *Adipoq* gene in 3T3-L1 adipocytes

**Figure 2:**
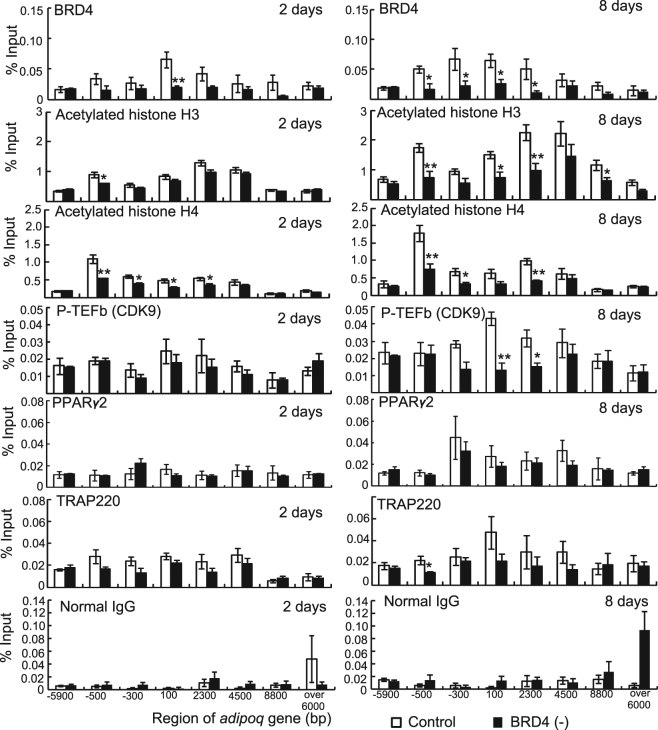
Effects of BRD4 depletion on binding of BRD4, acetylated histones, P-TEFb, PPARγ2, and TRAP220 around the *Adipoq* gene in 3T3-L1 adipocytes. Control or *Brd4* shRNA-expressing 3T3-L1 cells were treated with medium for differentiation. ChIP assays for BRD4, acetylated histone H3, acetylated histone H4, CDK9, PPARγ2, TRAP220, and normal IgG were performed at 2 and 8 days after differentiation. The data shown are means ± SEM of 6 wells per condition in a single experiment. *P < 0.05, **P < 0.01 versus the corresponding control cells by Student’s *t*-test.

To investigate whether BRD4 depletion reduces the levels of histone acetylation and recruitment of BRD4, P-TEFb, PPARγ2, and TRAP220 around the *Adipoq* gene, we performed ChIP assays using antibodies against these proteins in control or *Brd4* shRNA-expressing cells after induction of adipocyte differentiation (Fig. 2). Brd4 recruitment around the *Adipoq* gene was lower in *Brd4* shRNA-expressing cells than in control shRNA-expressing cells, with significant reductions observed at −100 bp at 2 days and from −500 to 2300 bp at 8 days. Compared with control shRNA-expressing cells, acetylation levels of histones around the *Adipoq* gene were lower in *Brd4* shRNA-expressing cells at −500 bp at 2 days, and at −500, 100, 2300, and 8800 bp at 8 days for histone H3. They were also lower from −500 to 2300 bp at 2 days, and at −500, −300, and 2300 bp at 8 days for histone H4. P-TEFb (CDK9) recruitment onto the transcribed region (100 and 2300 bp) was significantly lower in *Brd4* shRNA-expressing cells than in control shRNA-expressing cells. ChIP signals for PPARγ2 gradually increased from 2 to 8 days after differentiation stimulation, although the signals did not differ between control cells and *Brd4* shRNA expressing cells at 2 or 8 days. PPARγ2 and TRAP220 recruitment levels around the *Adipoq* gene were not remarkably altered, and a significant reduction by *Brd4* shRNA was only observed at −500 bp for TRAP220 at 8 days.

Together these results demonstrate that BRD4 depletion reduced acetylation of histone H3 and H4 and BRD4-P-TEFb recruitment around the *Adipoq* gene in 3T3-L1 adipocytes.

### Effects of depletion or overexpression of BRD4 on the expression of *Adipoq* and other insulin sensitivity-related genes in 3T3-L1 adipocytes

**Figure 3:**
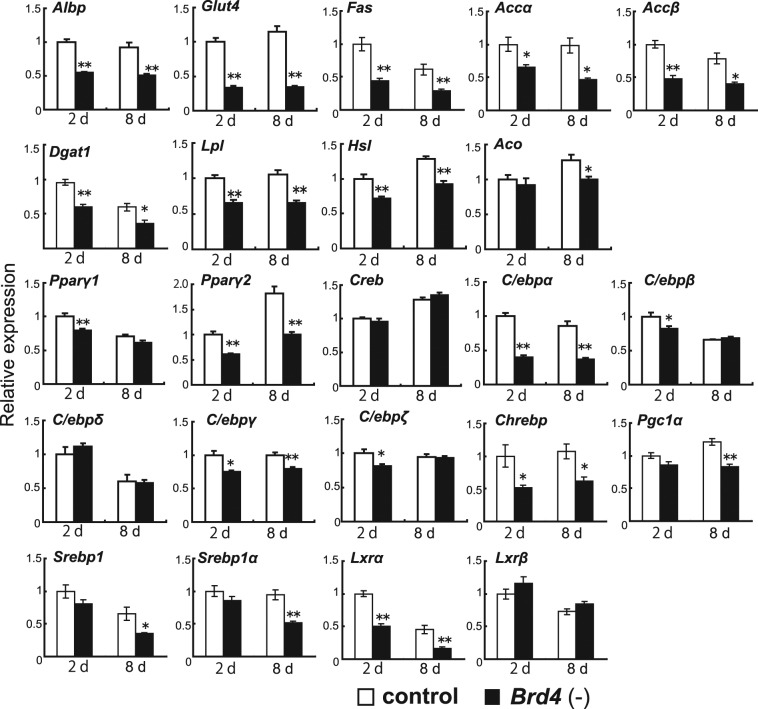
Effects of BRD4 depletion on expression of genes related to insulin sensitivity in 3T3-L1 adipocytes. Control or *Brd4* shRNA-expressing 3T3-L1 cells were treated with medium for differentiation and real-time RT-PCR analyses for *Albp*, *Glut4*, *Fas*, *Accα*, *Accβ*, *Dgat1*, *Lpl*, *Hsl*, *Aco*, *Pparγ1*, *Pparγ2*, *Creb*, *C/ebpα*, *C/ebpβ*, *C/ebpδ*, *C/ebpγ*, *C/ebpζ*, *Chrebp*, *Pgc1α*, *Srebp1*, *Srebp1α*, *Lxrα* and *Lxrβ* were performed at 2 and 8 days after differentiation stimulation. The data shown are means ± SEM of 6 wells per condition in a single experiment. *P < 0.05, **P < 0.01 versus the corresponding control cells by Student’s *t*-test.

To examine the effects of BRD4 protein depletion on the expression of genes related to insulin sensitivity, we performed real-time RT-PCR on *Brd4* shRNA-expressing and control shRNA-expressing cells (Fig. 3). ALBP is involved in fatty acid binding and incorporation, and GLUT4 is involved in glucose incorporation. Fatty acid synthase (FAS) and acyl-CoA carboxylase (ACC) are well-known rate-controlling enzymes for fatty acid synthesis. Lipoprotein lipase (LPL) is an enzyme required for hydrolysis of triacylglycerol derived from VLDL/chylomicron to fatty acids and glycerol, including in adipocytes. Diglyceride acyltransferase 1 (DGAT1) is an enzyme required for re-synthesis of triacylglycerol from fatty acids and glycerol in adipose tissues. Hormone-sensitive lipase (HSL) is an enzyme for generating free fatty acids from triacylglycerol. The mRNA levels of *Albp*, *Glut4*, *Fas*, *Accα*, *Accβ*, *Dgat1*, *Lpl*, and *Hsl* were lower in *Brd4* shRNA-expressing cells at 2 and 8 days after differentiation stimulation than in control shRNA-expressing cells. The expression levels of transcription factor genes for insulin sensitivity, including *Pparγ2*, *C/ebpα*, *C/ebpγ*, carbohydrate-responsive element-binding protein (*Chrebp*), and liver X receptor α (*Lxrα*), were also lower in *Brd4* shRNA-expressing cells at 2 and 8 days than in control shRNA-expressing cells. The mRNA levels of *Pparγ1*, *C/ebpβ* and *C/ebpζ* at 2 days and acyl-CoA oxidase (*Aco*), PPARγ coactivator 1α *(Pgc1α)*, sterol regulatory element-binding protein 1 (*Srebp*) and *Srebp1α* were lower in *Brd4* shRNA-expressing cells at 2 and 8 days after differentiation than in control shRNA-expressing cells.

Next, we examined whether treatment with (+)-JQ1 for 2 and 4 days after differentiation stimulation altered expression of genes related to insulin sensitivity in 3T3-L1 adipocytes. The mRNA expression of *Adipoq* and *Albp*, but not other genes (*Fas*, *Accα*, *Accβ*, *Dgat1*, *Lpl*, *Pparγ1*, and *Pparγ2*), was reduced by 500 nM (+)-JQ1 treatment for 2 days after differentiation stimulation. Treatment with (+)-JQ1 at 200 nM and 500 nM for 4 days reduced mRNA levels of *Adipoq*, *Albp*, *Accβ*, *Dgat1*, *Lpl*, *Pparγ1*, and *Pparγ2*. *Accα* mRNA was reduced by (+)-JQ1 treatment for 4 days at 200 nM, but not at 500 nM (Supplementary Fig. S1).

The binding of acetylated histone H3 to all regions tested and acetylated histone H4 from −500 to −2100 bp of *Glut4* gene were lower in *Brd4* shRNA-expressing cells at 2 days than in control shRNA-expressing cells. BRD4 binding at −1000 bp and 2100 bp and CDK9 binding at 1000 bp were lower in *Brd4* shRNA-expressing cells at 2 days than in control shRNA-expressing cells. The binding of acetylated histone H3 from −5000 bp to −1000 bp and from 1000 bp to 5000 bp and acetylated histone H4 at −1000 bp of the *Lxrα* gene were lower in *Brd4* shRNA-expressing cells at 2 days than in control shRNA-expressing cells. BRD4 binding from −2000 bp to 1 bp and 200 bp were lower in *Brd4* shRNA-expressing cells at 2 days than in control shRNA-expressing cells. CDK9 binding around the *Lxrα* gene showed no difference between the two groups (Supplementary Fig. S2).

**Figure 4:**
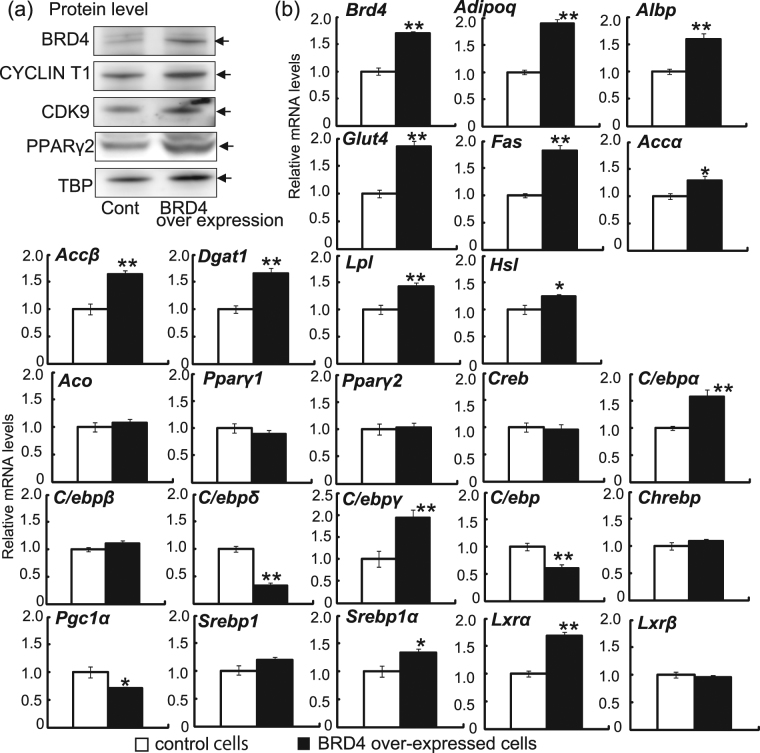
Effects of BRD4 overexpression on *Brd4* mRNA and protein levels and mRNA levels of genes related to insulin sensitivity in 3T3-L1 adipocytes. *Brd4*-overexpressing or control plasmid transfected 3T3-L1 cells were treated with medium for differentiation. Immunoblot analysis for BRD4, CYCLIN T1, CDK9, PPARγ2 and TBP and real-time RT-PCR analyses for *Brd4*, *Adipoq*, *Albp*, *Glut4*, *Fas*, *Accα*, *Accβ*, *Dgat1*, *Lpl*, *Hsl*, *Aco*, *Pparγ1*, *Pparγ2*, *Creb*, *C/ebpα*, *C/ebpβ*, *C/ebpδ*, *C/ebpγ*, *C/ebpζ*, *Chrebp*, *Pgc1α*, *Srebp1*, *Srebp1α*, *Lxrα* and *Lxrβ* were performed in samples at 2 days after differentiation. The data shown are means ± SEM of 6 wells per condition in a single experiment. *P < 0.05, **P < 0.01 versus the corresponding control cells by Student’s *t*-test.

Next, we used a retrovirus transfection system to construct a stable 3T3-L1 cell line overexpressing exogenous BRD4. At 2 days after differentiation stimulation, the cell line stably expressing exogenous BRD4 protein showed higher BRD4 mRNA and protein levels than control plasmid-expressing cells (Fig. 4). Protein expression levels of BRD4, CDK9, PPARγ2 and CYCLIN T1 in the stable BRD4-expressing cell line were 2.80-, 1.65-, and 1.16, 1.01-fold higher (normalised by TBP) compared with controls, respectively. *Adipoq* gene expression was higher in BRD4-overexpressing adipocytes than in control adipocytes at 2 days. The expression levels of genes related to insulin sensitivity, including *Albp*, *Glut4*, *Fas*, *Accα*, *Accβ*, *Dgat1* and *Lpl*, were higher in BRD4-overexpressing adipocytes than in control adipocytes. Expression of the *Hsl* gene was also higher in BRD4-overexpressing adipocytes than in control adipocytes. In contrast, β-oxidation-related genes, such as *Aco*, showed no changes in response to BRD4 overexpression. The expression levels of transcription factor genes, such as *C/ebpα*, *C/ebpγ*, *Srebp1α*, and *Lxrα*, but not *Pparγ1*, *Pparγ2*, *Creb*, *C/ebpβ*, *Chrebp*, *Srebp1* and liver X receptor β (*Lxrβ*), were higher in *Brd4*-overexpressing adipocytes than in control adipocytes. In contrast, the expression levels of *C/ebpδ*, *C/ebpζ* and *Pgc1α* were lower in *Brd4*-overexpressing adipocytes than in control adipocytes.

These results showed that BRD4 depletion reduced the expression of insulin sensitivity-related genes and BRD4 overexpression induced expression of these genes in 3T3-L1 adipocytes. In addition, BRD4 bound proximal regions of both *Glut4* and *Lxrα*, whereas CDK9 bound that of *Glut4*, but not *Lxrα*.

### Effects of *Brd4* depletion on *Adipoq* and fatty acid synthesis-related gene expression in mesenteric adipose tissues and ADIPOQ protein in serum during postnatal development of mice

**Figure 5:**
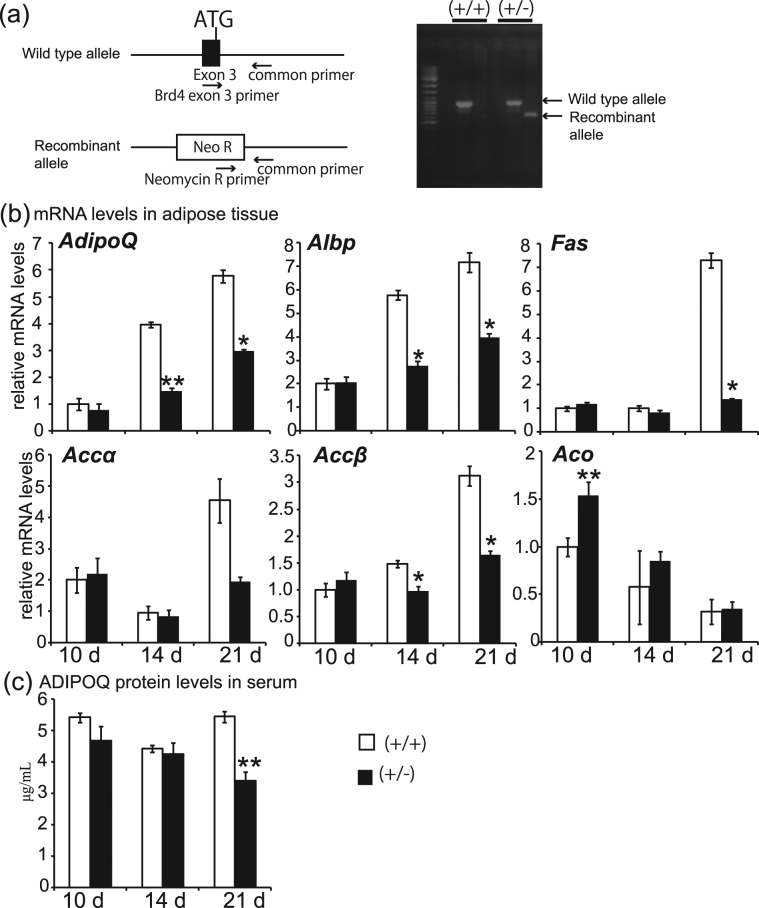
Effects of *Brd4* genetic depletion on *Adipoq* and fatty acid synthesis-related gene expression in mesenteric fat tissues and ADIPOQ protein in serum during postnatal development of mice. (**a**) Genotypes. (**b**) mRNA levels of *Adipoq* and genes related to fatty acid synthesis (*Albp*, *Fas*, *Accα* and *Accβ*) and fatty acid oxidation (*Aco*). (**c**) Serum ADIPOQ concentration. The data shown are means ± SEM for 5–11 mice per group in a single experiment. *P < 0.05, **P < 0.01 versus the corresponding control cells by Student’s *t*-test.

To investigate whether BRD4 regulates the induction of *Adipoq* and fatty acid synthesis-related genes in mice, we generated *Brd4* heterozygous (*Brd4*+*/−*) mice. The genetic strategy for generating *Brd4* heterozygous mice is shown in Fig. 5a. We performed real-time RT-PCR using mesenteric adipose tissue of wild-type and *Brd4* heterozygous mice during the suckling period (10 days after birth) and weaning periods (14 and 21 days after birth) (Fig. 5b). The expression levels of the *Adipoq*, *Albp*, and *Accβ* genes were lower at 14 and 21 days after birth in *Brd4* heterozygous mice than in wild-type mice at the same ages. The expression of *Fas* at 21 days after birth was significantly lower in *Brd4* heterozygous mice than in wild-type mice at the same age. The expression of *Aco* gene was temporarily higher at 10 days after birth in *Brd4* heterozygous mice than in wild-type mice at the same age, but the difference was not observed at other ages. Serum ADIPOQ protein levels were lower in *Brd4* heterozygous mice than in wild-type mice at 21 days, but not at 10 and 14 days. The expression levels of the other insulin sensitivity-related genes examined in Figs 3 and 4 were not remarkably altered (data not shown). Body weight and mesenteric and epididymal adipose tissue weights, but not liver weight, per 10 g of body weight, were lower in *Brd4* heterozygous mice at 20 weeks of age than in wild-type mice of the same age (Supplementary Fig. S3).

These data demonstrate that *Brd4* heterozygous mice showed lower levels of *Adipoq*, *Albp*, *Fas* and *Accβ* mRNAs, higher *Aco* mRNA levels during the suckling period in adipose tissues, lower ADIPOQ protein concentration in serum during the weaning period, and lower adipose tissue weights in adulthood.

## Discussion

Previous studies reported that *Adipoq* expression and adipocyte differentiation are regulated by PPARγ2^19,20^. In this study, we demonstrated direct *in vivo* binding of PPARγ2 and TRAP220, a component of the TRAP/DRIP/mediator complex also known to bind to PPARγ2^21^, to the promoter/enhancer and transcribed regions of the *Adipoq* gene during adipocyte differentiation of 3T3-L1 cells after differentiation stimulation. We demonstrated that BRD4 and P-TEFb were bound to the transcribed region of the *Adipoq* gene in 3T3-L1 adipocytes and that BRD4 depletion reduced P-TEFb binding, but not the binding of PPARγ2 and TRAP220, to the *Adipoq* gene in 3T3-L1 adipocytes. Together this suggests that BRD4 recruits P-TEFb strongly onto the *Adipoq* gene and BRD4 directly regulates the *Adipoq* gene by recruiting P-TEFb to the transcribed region of the gene. We also demonstrated that PPARγ2 binding around the *Adipoq* gene was not significantly reduced by *Brd4* shRNA, although BRD4 and CDK9 binding around the gene were reduced by *Brd4* shRNA. These results indicate that reduction of the *Adipoq* gene expression in 3T3-L1 adipocytes by *Brd4* shRNA was associated with BRD4-P-TEFb binding level, but less so with PPARγ2 binding level, to the *Adipoq* gene.

Whether BRD4 directly regulates the *Adipoq* gene in adipocytes remains unclear. In this study, we demonstrated that treatment with (+)-JQ1, an inhibitor of the association between BET family members including BRD4 and acetylated histones^22^, for 2 and 4 days after differentiation stimulation markedly reduced *Adipoq* mRNA levels and lipid droplet accumulation in 3T3-L1 adipocytes. In addition, we revealed in shRNA experiments that BRD4 regulates ADIPOQ expression by inducing P-TEFb binding around acetylated histones on the transcribed region of the gene. The results of (+)-JQ1 treatment with 3T3-L1 adipocytes support the results of *Brd4* shRNA. However, (+)-JQ1 also inhibits other BET family members such as BRD2 and BRD3. Indeed, deficiency of BRD2 in mice by gene targeting induces obesity^23^. Thus, BRD2 may reduce lipid droplet accumulation and BRD4 may increase lipid droplet accumulation. In this study, we have shown that BRD2 protein expression was not only induced during adipocyte differentiation, but was also reduced by *Brd4* shRNA expression at a late stage (8 days after differentiation), but not at an early stage (2 days after differentiation) of adipocyte differentiation in 3T3-L1 adipocytes. It has been reported that expression of insulin sensitivity-related genes is reduced at a late stage of 3T3-L1 adipocyte differentiation^24^. In addition, BRD2 inhibits adipogenesis by repressing PPARγ2 and C/EBPα expression^25^. Combined with this evidence, BRD2 may inhibit adipogenesis, in particular at the late stages of adipogenesis, and BRD4 may maintain BRD2 expression at later stages. Therefore, regulation of the balance between BRD4 and BRD2 activities may inhibit metabolic diseases, such as obesity, type 2 diabetes, and related complications. Thus, the regulation of adipogenesis by BET family members including BRD4 and BRD2 should be examined in further studies using shRNAs.

(+)-JQ1 treatment at 500 nM for 2 days and at 200 nM for 4 days reduced expression of *Adipoq*, but not of *Pparγ2*. Thus, inhibition of the association between acetylated histones and BET family proteins including BRD4 by (+)-JQ1 after differentiation stimulation reduces gene expression of *Adipoq* rather than *Pparγ2*. Furthermore, we found that BRD4 directly binds to chromatin around the *Adipoq* gene and that *Brd4* shRNA reduced BRD4 and P-TEFb binding, but had a smaller effect on PPARγ2 binding around the *Adipoq* gene in 3T3-L1 adipocytes. In addition, we showed that BRD4 bound to chromatin near *Glut4* and *Lxrα* genes in 3T3-LI adipocytes. These results indicate that BRD4 directly binds to chromatin around the *Adipoq*, *Glut4* and *Lxrα* genes and that BRD4 may directly regulate expression of these genes in adipocytes. Furthermore, BRD4 would regulate adipogenesis because 500 nM (+)-JQ1 treatment for 4 days reduced *Pparγ2* expression and reduction of BRD4 protein by shRNA also reduced expression of *Pparγ2* and *C/ebpα* genes in 3T3-L1 adipocytes, although reduction of gene expression by shRNA and (+)-JQ1 was greater for *Adipoq* than *Pparγ2*. Taken together, these findings indicate that it is the decrease of BRD4 binding around the *Adipoq* gene, rather than that of adipogenesis triggered by PPARγ2, that contributes to repression of *Adipoq* gene expression by BRD4 shRNA and BET inhibitor. However, the contribution of the direct effect of BRD4 and the effect of inhibiting adipogenesis by BRD4 on expression of genes related to insulin sensitivity in the adipocytes should be examined in further studies.

We demonstrated that *Adipoq* and *Glut4* genes were regulated by both BRD4 and P-TEFb, whereas the *Lxrα* gene was regulated by BRD4 alone. A recent study demonstrated that Pol II activation of cell cycle genes has several patterns, namely, BRD4-dependent genes, P-TEFb-dependent genes, and BRD4/P-TEFb-dependent genes in NIH3T3 cells^26^. These insights indicate that associations between BRD4 and acetylated histones and those between BRD4 and P-TEFb should be determined for each gene by ChIP assays. A previous study showed that BRD4 itself as well as P-TEFb activates Pol II by inducing direct phosphorylation of Ser 2 in CTD-Pol II^27^. Thus, Pol II on P-TEFb-independent genes in 3T3-L1 adipocytes may be phosphorylated by BRD4. However, this hypothesis should be examined in further studies. In addition, future studies should examine whether BRD4 directly binds to acetylated histones and to P-TEFb in 3T3-L1 adipocytes.

In this study, we demonstrated that the acetylated histone H3 and H4 levels around the *Adipoq* gene, as well as the *Adipoq* mRNA levels and BRD4-P-TEFb binding, were reduced by *Brd4* shRNA expression. It is most likely that BRD4 targets the increased acetylated histones around the *Adipoq* gene during adipocyte differentiation. *Adipoq* gene expression is regulated by the histone acetyltransferase (HAT) CREB-binding protein^28^. In addition, another HAT, GCN5, acetylates histones in the transcribed region^29^. Thus, it is possible that BRD4 recruits these HATs onto various target genes. However, there is no evidence for whether BRD4 associates with HATs. This aspect should be examined in further studies.

We demonstrated that the transcription of insulin sensitivity-related genes, such as *Albp*, *Glut4*, *Fas*, *Accα*, *Accβ*, *Dgat1*, *Lpl*, as well as *Adipoq*, is reduced by BRD4 inhibition. Treatment with (+)-JQ1 reduced mRNA levels of *Albp*, *Accβ*, *Dgat1* and *Lpl*. In addition, BRD4 depletion by shRNA and inhibition by (+)-JQ1 reduced lipid droplet accumulation in 3T3-L1 adipocytes, which was also evident in *Brd4* heterozygous mice. We additionally showed that the gene expression levels of the insulin-dependent transcription factor *Srebp1* and carbohydrate-dependent transcriptional factor *Chrebp* ^30,31^ were lower in BRD4-depleted adipocytes. It should be noted that the expression of PPARγ2 protein was reduced in BRD4-depleted adipocytes compared with non-depleted adipocytes. This result indicates that insulin sensitivity genes except for *Adipoq* may be reduced with a decrease in *Pparγ* mRNA. However, the induction of BRD4 in 3T3-L1 adipocytes increased expression of insulin sensitivity-related *Adipoq*, *Albp*, *Fas* and *Accβ* but did not change *Pparγ2* expression, and heterozygosity of *Brd4* in mice decreased expression of these insulin sensitivity-related genes. These results suggest that BRD4 affects the expression of insulin sensitivity-related genes, but has less of an effect on *Pparγ2* expression *in vivo* and *in vitro*. Furthermore, they indicate that BRD4 is important for lipid droplet accumulation in adipose tissues by regulating the expression of genes related to insulin sensitivity. In this study, we demonstrated that BRD4 binds regions around *Adipoq*, *Glut4* and *Lxrα* genes in 3T3-L1 adipocytes. Whether BRD4 regulates each gene related to insulin sensitivity in adipocytes directly should be examined in future studies.

In conclusion, our study demonstrated that: (1) BRD4 regulates *Adipoq* gene expression by recruiting P-TEFb onto acetylated histones in the transcribed region of the gene; (2) BRD4 regulates adipocyte differentiation and lipid droplet accumulation by regulating the expression of genes related to insulin sensitivity; and (3) BRD4 is important for the development of adipose tissues and the expression of *Adipoq* and fatty acid synthesis-related genes during postnatal development. These findings suggest that BRD4 is important for maintaining the homeostasis of adipose tissue and fat metabolism in adipocytes.

## Materials and Methods

### Cell culture

The 3T3-L1 preadipocyte cells (American Type Culture Collection, Manassas, VA) were cultured at 37 °C in Dulbecco’s modified Eagle’s medium (DMEM) containing 10% donor bovine serum (Invitrogen, Carlsbad, CA), 2 mM glutamine, 20 mM HEPES (pH 7.4), 50 U/ml penicillin, and 50 µg/ml streptomycin sulfate in a humidified atmosphere of 5% CO_2_. The control and *Brd4* shRNAs, which were expressed as siRNAs in the cells, were constructed in the pSUPERRNAi vector (Oligoengine, Seattle, WA)^16^. The *Brd4* shRNA sequence corresponded to positions+604 to+623 from the translation start site: 5′-GATCCCCGACTTCTCCGCAGATTCAAGAGATCTGCGGAGAATTGATGCTTTTTGGAAA-3′. The control shRNA was a shuffled version of the above sequence: 5′-GATCCCCATGCACGTGCACATATCCCTTCAAGAGAGGGATATGTGCACGTGCATTTTTTGGAAA-3′. For preparation of BRD4-overexpressing cells, a mouse *Brd4* cDNA (Unigene ID Mm. 253518) was inserted into the MSCVneo vector (Takara Bio, Shiga, Japan). The resulting constructs were separately transfected with Gag-Pol and pEco into HEK293T packaging cells with Lipofectamine 2000 (Invitrogen) according to the manufacturer’s instructions, and the cells were cultured for 2 days with DMEM containing 10% donor bovine serum. Supernatants were collected 2 days after transfection as virus-containing media. Cells were transduced with the virus-containing media mixed with 6 µg polybrene (Sigma-Aldrich) by centrifugation (1,000 × *g*) for 2 h at 32 °C and continuously cultured for 2 days. The transduced cells were then selected with puromycin (Sigma-Aldrich) for *Brd4*/control shRNA-expressing cells or G418 (Sigma-Aldrich) for *Brd4*-/empty-MSCVneo-transfected cells for 7 days. The growth of *Brd4* shRNA-expressing 3T3-L1 cells tended to be reduced by approximately 20–30% during 3 days of culture compared with control cells, as previously reported for NIH3T3 cells in asynchronised conditions^17^. The cell growth of *Brd4*-MSCVneo-transfected and empty-MSCVneo-transfected cells did not differ. After reaching 60–80% confluence, the cells were trypsinised, and large numbers of cells (2.7 × 10^4^/cm^2^) were seeded and cultured in differentiation medium (DMEM supplemented with 10% FBS, 0.5 mM 3-isobutyl-1-methylxanthine, 2 µM dexamethasone, and 1.7 µM insulin). After 48 h of stimulation (defined as day 0 in this study), untreated 3T3-L1 cells and the transfected cells were cultured in DMEM containing 10% FBS. The cell numbers between the control and *Brd4* shRNA-expressing 3T3-L1 cells, and between the empty-MSCVneo-transfected and *Brd4*-MSCVneo-transfected cells did not differ after the stimulation of differentiation.

For treatment with (+)-JQ1, 3T3-L1 cells were cultured in DMEM containing 10% FBS with (+)-JQ1 at concentrations of 0, 200 and 500 nM for 4 days, just after differentiation stimulation.

### Animals

BAC clones containing the murine *Brd4* locus were isolated using the Do-It-Yourself PCR screening kit (Mouse ES BAC, Release I) (Incyte, Wilmington, DE). A targeting vector was constructed by placing the NcoI (7.5-kb) and HindIII-XhoI (3.4-kb) fragments upstream and downstream of the SA-IRES-linked selection cassette containing the neomycin resistance gene, respectively. Cells were electroporated with the linearized targeting vector and selected in the presence of G418. Clones with a disrupted *Brd4* gene were identified by Southern blot analysis using probes external to the recombination construct as described previously^32^. The *Brd4* heterozygous (*Brd4*+*/−*) mice were generated in a NIH animal facility^33^. The mice were derived from C57BL/6 J mice and bred with wild-type mice purchased from Japan Oriental Yeast Co. Ltd. (Shizuoka, Japan). The *Brd4* heterozygous mice at 10–21 days after birth were divided into three groups: the first group (control, n = 10; *Brd4*+/−, n = 10) analysed at 10 days after birth; the second group (control, n = 11; *Brd4*+/−, n = 5) analysed at 14 days after birth; and the third group (control, n = 7; *Brd4*+/−, n = 7) analysed at 21 days after birth. All mice were euthanised by decapitation between 10:00 and 11:00 at 10, 14, and 21 days, respectively, after birth, and their serum, tail, and mesenteric adipose tissues were collected. The animals were genotyped using their tail DNA by PCR with the following primers: *Brd4* exon 3 primer: 5′-GGACTAGAAACCTCCCAAATGTCTACAA-3′; neomycin R primer: 5′-TGAAGAGCTTGGCGGCGAATGGG-3′; and common primer: 5′-CCTGTGTGCACTTGCTCCCGAGGAGAGA-3′. The PCR products were subjected to 1% agarose gel electrophoresis and stained with ethidium bromide. The experimental procedures in the present study were approved by the Institutional Animal Care and Use Committee (IACUC) of the University of Shizuoka in accordance with a guideline for animal experiments of the Ministry of Education, Culture, Sports, Science and Technology in Japan.

### Real-time RT-PCR

Total RNA was extracted by an acidified guanidine thiocyanate method as described previously^34^. The total RNA samples (2.5 µg) were converted into cDNA by a reverse-transcription reaction using SuperScript II reverse transcriptase (Invitrogen) according to the manufacturer’s instructions. For quantitative analysis of the mRNA levels, PCR was performed using a Light-Cycler Instrument System (Roche Molecular Biochemicals, Penzberg, Germany). The real-time PCR amplifications were carried out using gene-specific primers, cDNA, and SYBR Premix Ex Taq (Takara Bio). The CT values of each gene detected by real-time RT-PCR were converted into signal intensities based on the delta–delta CT method that recognises a difference of one CT value as a two-fold difference between samples. The RNA levels were normalised by the levels of 18 S rRNA (3T3-L1 cells) or 12 S rRNA (mouse adipose tissues) obtained from the same sample. The 18 S rRNA and 12 S rRNA levels were not altered by shRNA treatment and adipocyte differentiation, or by postnatal development in adipose tissues, respectively. The primer sequences are shown in Supplementary Table S1.

### Immunoblot

Total cell proteins extracted in RIPA buffer and cell extracts were separated by 10% SDS-PAGE and subjected to immunoblotting as previously described^35^. The primary antibodies used in this study were as follows: anti-PPARγ2 (#MAB3630, Chemicon, Temecula, CA); anti-BRD4, which was generated against the BRD4 C-terminal peptide (CFQSDLLSIFEENLF) by a custom antibody service (Sigma-Aldrich); anti-BRD2 (#5848, Cell Signalling Technology, Danvers, MA); anti-TRAP220 (#sc-8998, Santa Cruz Biotechnology, Dallas, TX); anti-CDK9 (#sc-8338, Santa Cruz Biotechnology); and anti-CYCLIN T1 (#ab2098, Abcam, Cambridge, UK). The ADIPOQ protein levels in medium and serum were measured by ELISA (Mouse Adiponectin ELISA Kit; CycLex, Nagano, Japan).

### ChIP assay

ChIP assays were performed as previously described^15^. The 3T3-L1 adipocytes were incubated with fixation solution (1% formaldehyde, 4.5 mM HEPES pH 8.0, 9 mM NaCl, 0.09 mM EDTA, 0.04 mM EGTA) in culture medium for 15 min at 37 °C and the reaction was terminated by the addition of glycine to a final concentration of 150 mM. After being washed with 2% bovine serum and 0.05% NaN_3_ in 1 × PBS(−), the samples were sonicated in SDS lysis buffer [50 mM Tris-HCl pH 8.0, 10 mM EDTA pH 8.0, 1% SDS, cOmplete Mini (one tablet/10 mL SDS lysis buffer, Roche Molecular Biochemicals)] until the DNA size of samples was under 500 bp. One-tenth of the volume of the sample was diluted in dilution buffer (50 mM Tris-HCl pH 8.0, 167 mM NaCl, 1.1% Triton X-100, 0.11% sodium deoxycholate) and stored at 4 °C as the input fraction. The remaining solutions were divided into several aliquots and incubated with 1 µg of the following antibodies: anti-acetyl-histone H3 at K9 and K14 (#06–599, Millipore, Billerica, MA); anti-acetyl-histone H4 at K5, K8, K12, K16 (#06–598, Millipore); anti-PPARγ2 (MAB3630, Chemicon); anti-BRD4 from the custom antibody service (Sigma-Aldrich); anti-TRAP220 (#sc-8998, Santa Cruz Biotechnology); anti-CDK9 (#sc-8338, Santa Cruz Biotechnology); and control rabbit IgG (#15006, Sigma-Aldrich) for 8–12 h at 4 °C. The protein-DNA-antibody complexes were immunoprecipitated by protein G sepharose (GE Healthcare, Little Chalfont, UK) containing 100 µg/ml salmon sperm DNA (Sigma-Aldrich) and 1% BSA in RIPA buffer (50 mM Tris-HCl pH 8.0, 1 mM EDTA, 1% Triton X-100, 0.1% SDS, 0.1% sodium deoxycholate) with 150 mM NaCl. Protein G sepharose was washed twice in RIPA buffer/150 mM NaCl, four times in RIPA buffer/500 mM NaCl, twice in LiCl buffer (10 mM Tris-HCl pH 8.0, 0.25 M LiCl, 1 mM EDTA pH 8.0, 0.5% NP-40, 0.5% sodium deoxycholate) and twice in TE buffer (10 mM Tris-HCl pH 8.0, 1 mM EDTA). Protein-DNA-Protein G sepharose complexes obtained by immunoprecipitation, and input fractions were dissociated in 200 µl ChIP direct elution buffer (10 mM Tris-HCl pH 8.0, 5 mM EDTA, 0.5% SDS, 300 mM NaCl) at 65 °C overnight. After removal of RNA and protein by the treatment with RNase A and proteinase K and phenol/chloroform extraction, the precipitated DNA was subjected to real-time PCR using primers corresponding to the indicated sites in the promoter/enhancer, transcription, and downstream regions (Supplementary Table S2). The CT values of the ChIP and input signals detected by real-time PCR were converted into signal intensities by the delta–delta CT method. All ChIP signals were normalised by the corresponding input signals.

### Oil red O staining

The 3T3-L1 adipocytes were cultured in 6-well plates for 4 or 5 days after differentiation stimulation. After removal of the culture medium, the cells were washed twice with PBS and fixed with 10% formalin/PBS. After three washes with PBS, the cells were incubated in 60% isopropanol for 10 min and stained with 1.8% Oil red O in 60% isopropanol for 10 min. After three further washes, the cells were observed under a light microscope.

## Electronic supplementary material

Supplementary Information
