# Supplementary material for: BRD4 regulates adiponectin gene induction by recruiting the P-TEFb complex to the transcribed region of the gene
Source: Sci Rep. 2017 Sep 20;7:11962. doi: 10.1038/s41598-017-12342-2 (PMC5607256; doi:10.1038/s41598-017-12342-2)
Supplement: Supplementary file 1 — Supplementary Information [file 41598_2017_12342_MOESM1_ESM.pdf]

Supplementary materials

Manuscript title: BRD4 regulates adiponectin gene induction by recruiting the P-TEFb complex to the transcribed region of the gene

Author name: Naoko Sakurai, Yuko Imamochi, Takuya Inoue, Natsuyo Hariya, Musashi Kawamura, Masami Yamada, Anup Dey, Akira Nishiyama, Takeo Kubota, Keiko Ozato, Toshinao Goda Kazuki Mochizuki

List of Supplementary materials

S1 Table
S2 Table
S1 Fig
S2 Fig
S3 Fig

S1 Table. Primer sets used for real time RT-PCR.

Gene		Sequence
18S rRNA	sense	5'-GGACACGGACAGGATTGACAGA-3'
	anti-sense	5'-TAGCATGCCAGAGTCTCGTTCG-3'
12S rRNA	sense	5'-AATCGATAAACCCCGCTCTAC-3'
	anti-sense	5'-TTCATTGGCTACACCTTGACC-3'
AdipoQ	sense	5'-AGGCCGTTCTCTTCACCTACGA-3'
	anti-sense	5'-GACTTGGTCTCCCACCTCCAGA-3'
Albp	sense	5'-CAAGCCCAACATGATCATCAGC-3'
	anti-sense	5'-CACGCCCAGTTTGAAGGAAATC-3'
Brd4	sense	5'-AATCATTCGCAGCGAGCCTTT-3'
	anti-sense	5'-TTGGGTGCCACTGGTGTTTTT-3'
Glut4	sense	5'-CTACCCTGTGGCCACTGCTTCT-3'
	anti-sense	5'-GGTATCTGGGGCGCGCAGGACA-3'
Fas	sense	5'- GTGTGGACATGGTCACAGATG-3'
	anti-sense	5'-GACCGCTTGGGTAATCCATA-3'
Accα	sense	5'-TTACATCCGCTTGGCTGAG-3'
	anti-sense	5'-TCCTCCCGCTTCTTCAACT-3'
Accβ	sense	5'-GCGAAAACCCAGATGAGG-3'
	anti-sense	5'-GTTCTTGTTGCTGCGGAAG-3
Dgat	sense	5'-GGTTAACCTGGCCACAATCA-3'
	anti-sense	5'-AGCAAACACGGAACCCACT-3'
Lpl	sense	5'-TCAGAGCCAAGAGAAGCAGCAA-3'
	anti-sense	5'-TTGTGTTGCTTGCCATCCTCA-3'
Hsl	sense	5'-GTGGCGAAAAGGCAAGATCAA-3'
	anti-sense	5'-TCATCGTGCGTAAATCCATGC-3'
Aco	sense	5'-TCATCGTGCGTAAATCCATGC-3'
	anti-sense	5'-AAATCCCAAGCAGCCCAATTC-3'
Pparγ1	sense	5'-GAGGACGCGGAAGAAGAGACCT-3'
	anti-sense	5'-CAGTGGTTCACCGCTTCTTTCA-3'
Pparγ2	sense	5'-GATTCTCCTGTTGACCCAGAGCA-3'
	anti-sense	5'-CATAGGCAGTGCATCAGCGAAG-3'
Creb	sense	5'-ACGGATGGACAGCAGATTCTA-3'
	anti-sense	5'-GCTGTGCGAATCTGGTATGTT-3'
C/ebpα	sense	5'-AAGCCAAGAAGTCGGTGGACAA-3'
	anti-sense	5'-CACGTTGCGTTGTTTGGCTTT-3'
C/ebpβ	sense	5'-ATGCAATCCGGATCAAACGTG-3'
	anti-sense	5'-CAACCCCGCAGGAACATCTTTA-3'
C/ebpδ	sense	5'-GCACGGCCTGTTGTACAGAAAA-3'
	anti-sense	5'-CACTTTGGGCAGGGATTTGAA-3'
C/ebpγ	sense	5'-AGCATTTGGTTTGGGGGAGAA-3'
	anti-sense	5'-ATGGCCCCATTGGCAGTTATT-3'
C/ebpζ	sense	5'-GACATGTTCCAAGCAAGCGATG-3'
	anti-sense	5'-AATGAATGGCGGCATCTGTGT-3'
Chrebp	sense	5'-CCTGAAGACCCTAAGACCAAGA-3'
	anti-sense	5'-TAAGCCATGCACCTTGACAG-3'
Pgc1α	sense	5'-GGAATGCACCGTAAATCTGC-3'
	anti-sense	5'-CAGGTGTAACGGTAGGTGATGA-3'

Srebp1	sense	5’-GCTGCTTCTAACCTGGCACTAA-3’
	anti-sense	5’-CCAGTGTTGCCATGGAGATAG-3’
Srebp1α	sense	5’-AGTGGCAAAGGAGGCACTAC-3’
	anti-sense	5’-GATAGCAGGATGCCAACAGC-3’
Lxrα	sense	5’-CGCGACAGTTTTGGTAGAGG-3’
	anti-sense	5’-CTCCAGCCACAAGGACATC-3’
Lxrβ	sense	5’-GCTCTGCCTACATCGTGGTC-3’
	anti-sense	5’-CTCATGGCCCAGCATCTT-3’

S2 Table Primer sets used for ChIP assays.

Region on the *Adipoq* gene	Sequence
-5900	ATGGCTCAGTGGTTAAGAGCA
	GATGGTTGTGAGCCTTCATGT
-500	TGCATGCATATTTGCACACCAA
	TCAATTCCCAGCACCCACAGTA
-300	ATGCCTGAACCACACAGCTTCA
	AGGGGTCAGGAGACCTCCCTTT
-100	TTCCCAGACCCAAGCTGGATTA
	CAACCCAGTCAAGGCCAATAGC
100	GGCCACTTTCTCCTCATTTCT
	TTTGGTGTCGTCAGATCCACT
2300	TGATTGGGTTGTGCCATTGTG
	GGCATTTGCCCAATGTGTATGA
4500	TCCCCATGGAAAAGATTGGTG
	CCGCCATTGCTCTGAGACTTTT
8800	TTCCTCTTAATCCTGCCCAGT
	ATCCAACCTGCACAAGTTCC
over 6000	AAAGGAAGGAAGGAAGGAAGG
	TGAGCTTTGCCCTTTTATGC

Region on the *Glut4* gene	Sequence
-5000	CTGCTACCTTGTGGGACAAA
	CTCCTGACATGTGCACACAA
-2000	ATGGGTAGAGGCAAGAAAG
	TTCTACCCAGAGTGTTGGGA
-1000	AAGGAACTTGAAGGAGGTCC
	TAACCTACAACCCAGCCCTC
-500	AGATGCGTGGAAAGAAAGG
	GAGATGATCCAAGGGACCAA
1	TTGTGAAGGGCGTGTCCTAT
	AAAGATGCGGAGAGCTGAA
1000	GACCTAAAAGGCTACCCCAA
	AAGTTCTCTCAGCTTGCTCCA
2100	CTATGCTGGCCAACAATGTC
	AGGAACCGTCCAAGAATGAG
5000	TACCTCCAGGTTGAAGGAAC
	AAGAATCACACAGGGGGAAT
over5000	AATCAGTGAGGACTCCAACC
	CCTTCTGGAGTTGCTGCATTA

Region on the *Lxrα* gene	Sequence
-5000	CCCCAAGGTATCTGGAGATT
	GCTTTAGAGCCCACAAAGTG
-2000	AGGAAGGAACCCATGAGAA
	CATAAGGTCATCCCCTCACA
-1000	AGGAACTGGGAGTGGACAG
	TCTTTAAGATGGGGACGATG

-500	CCCCTTGGGAGATTAAATCA<br>ATAGCTTTCCTGCCTTGCTTC
1	AGTCCTTCTGTCAGAGCAAA<br>TACCAAAACTGTCGCGTTTC
1000	CTGAAGTGTCCTGGGTTCAA<br>CCAGGATTAAAGGCATGCAG
2000	GGGTGTCTTGATATGCAAAG<br>AGCCACAGCTCATGATTTCA
5000	TGGGGTTCAGGTGCTTATAC<br>GCATCAACTCCTCAGATACT
over5000	GGCAGCCACAATGTCTTGTT<br>CCCGGCAGCATGTATTTATT

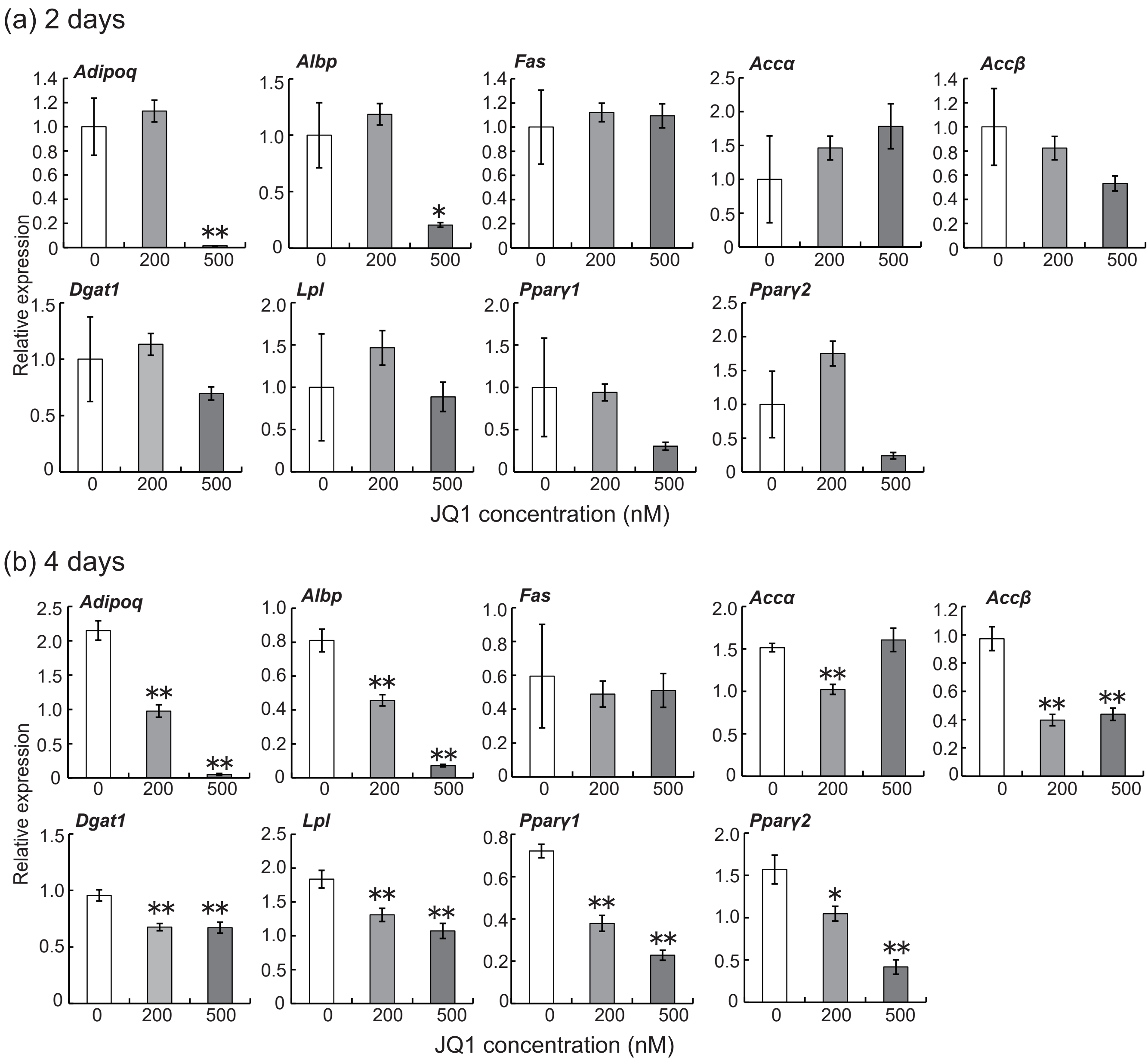
(a) 2 days
Adipoq
Albp
Fas
Accα
Accβ
Dgat1
Lpl
Pparγ1
Pparγ2
Relative expression
JQ1 concentration (nM)
0
200
500
(b) 4 days
Adipoq
Albp
Fas
Accα
Accβ
Dgat1
Lpl
Pparγ1
Pparγ2
Relative expression
JQ1 concentration (nM)

S1 Fig

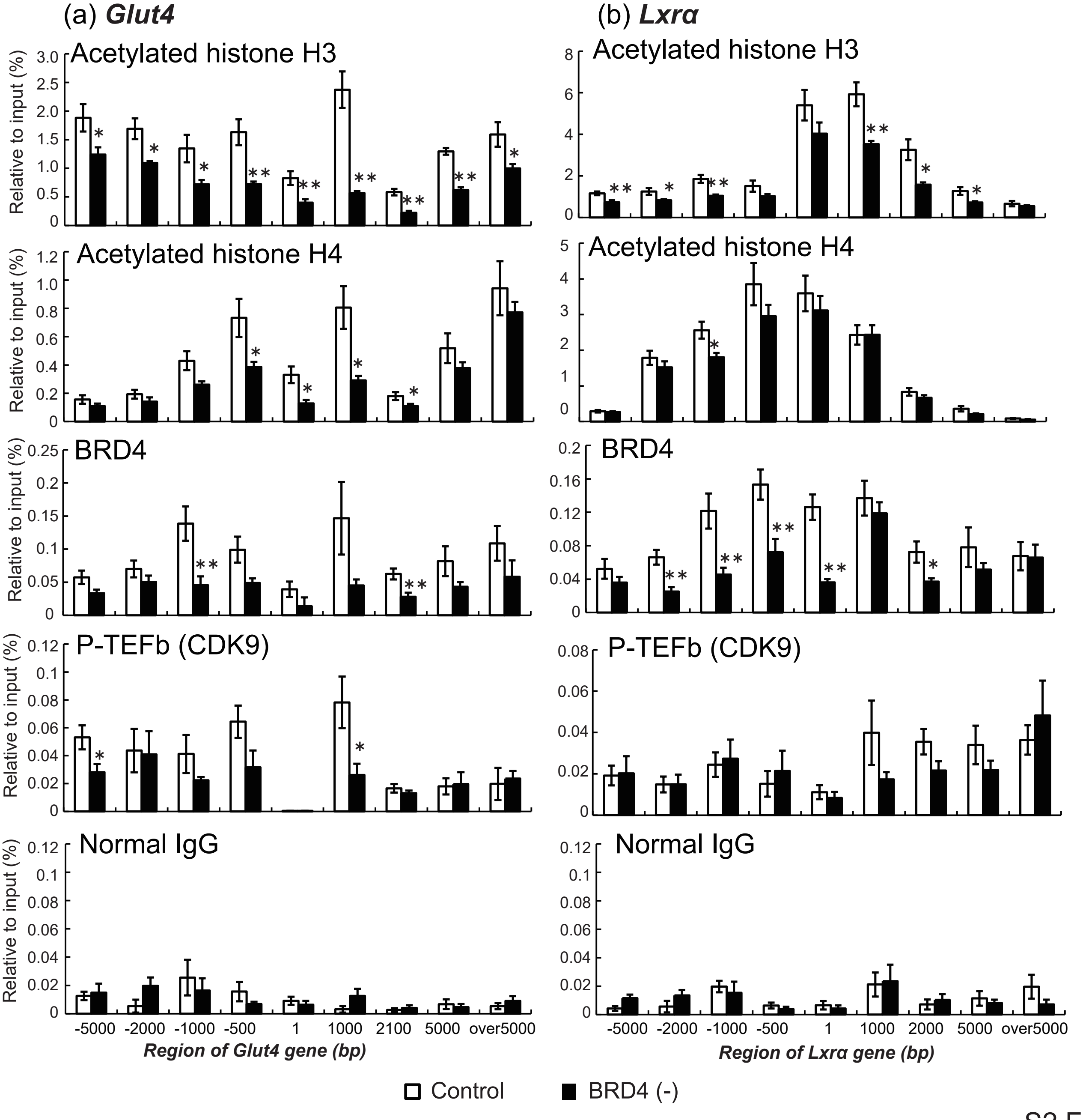

(a) Glut4
(b) Lxrα
Acetylated histone H3
Acetylated histone H4
BRD4
P-TEFb (CDK9)
Normal IgG
Relative to input (%)
-5000
-2000
-1000
-500
1
1000
2100
2000
5000
over5000
Region of Glut4 gene (bp)
Region of Lxrα gene (bp)
Control
BRD4 (-)

S2 Fig

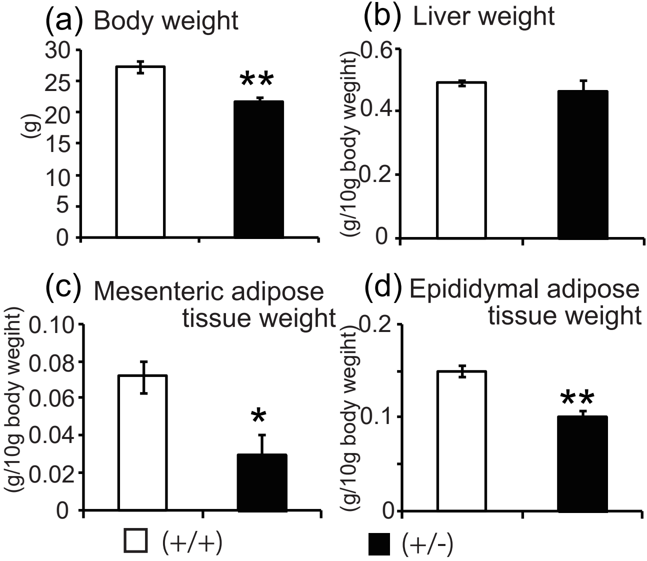

(a) Body weight
30
25
20
15
10
5
0
(g)
**
(b) Liver weight
0.6
0.4
0.2
0
(g/10g body weginht)
(c) Mesenteric adipose tissue weight
0.10
0.08
0.06
0.04
0.02
0
(g/10g body weginht)
*
(d) Epididymal adipose tissue weight
0.2
0.1
0
(g/10g body weginht)
**
(+/+)
(+/-)

S3 Fig

Supplementary Figure Legends

Supplementary Figure S1. Effects of inhibition of the BET family on mRNA levels of genes related to insulin sensitivity in 3T3-L1 adipocytes. 3T3-L1 adipocytes were treated with (+)-JQ1 for 2 and 4 days after adipocyte differentiation stimulation and mRNA levels of genes related to insulin sensitivity determined. he data shown are means ± SEM of six wells per condition in a single experiment. * $P < 0.05$, ** $P < 0.01$ versus the corresponding control cells by Dunnett's test based on analysis of variance.

Supplementary Figure S2. Effects of BRD4 depletion on the binding of BRD4, acetylated histones H3 and H4, and P-TEFb (CDK9) around *Glut4* and *Lxrα* genes in 3T3-L1 adipocytes. Control or *Brd4* shRNA-expressing 3T3-L1 cells were treated with medium for differentiation. Cells were collected and ChIP assays performed using antibodies against acetylated histone H3, acetylated histone H4, BRD4 and CDK9, and normal IgG as a control, at 2 days after differentiation. (a) *Glut4*. (b) *Lxrα*. The data shown are means ± SEM of six wells per condition in a single experiment. * $P < 0.05$, ** $P < 0.01$ versus the corresponding control cells by Student's *t*-test.

Supplementary Figure S3. Weights of body, liver and adipose tissues in 20-week-old wild-type and *Brd4* (+/−) mice. (a) Body weight. (b) Liver weight. (c) Mesenteric adipose tissue weight. (d) Epididymal adipose tissue weight. The data shown are means ± SEM for wild-type mice (n = 6) and *Brd4* (+/−) mice (n = 3) in a single experiment. * $P < 0.05$, ** $P < 0.01$ versus the corresponding control tissue or cells by Student's *t*-test.
